# The “Three in One” Bone Repair Strategy for Osteoporotic Fractures

**DOI:** 10.3389/fendo.2022.910602

**Published:** 2022-06-09

**Authors:** Xiao Chen, Yan Hu, Zhen Geng, Jiacan Su

**Affiliations:** ^1^ Department of Traumatic Orthopedics, First Affiliated Hospital of Navy Medical University, Shanghai, China; ^2^ Institute of Translational Medicine, Shanghai University, Shanghai, China

**Keywords:** Three in one, osteoporotic fractures, repair, treatment, strategy

## Abstract

In aging society, osteoporotic fractures have become one major social problem threatening the health of the elderly population in China. Compared with conventional fractures, low bone mass, bone defect and retarded healing issues of osteoporotic fractures lead to great difficulties in treatment and rehabilitation. Addressing major concerns in clinical settings, we proposed the “three in one” bone repair strategy focusing on anti-osteoporosis therapies, appropriate bone grafting and fracture healing accelerating. We summarize misconceptions and repair strategies for osteoporotic fracture management, expecting improvement of prognosis and clinical outcomes for osteoporotic fractures, to further improve therapeutic effect and living quality of patients.

## 1 Introduction

Osteoporosis is a systemic metabolic bone disease characterized by decreased bone strength and increased risk of fracture. Osteoporotic fractures refer to fractures caused by force that does not usually cause fractures (1). Data from the first epidemiological survey of osteoporosis among residents in China show that the prevalence of osteoporosis among people over 50 years old is 19.2%, and the prevalence rate over 65 years old is 32%. Among them, the prevalence of females is 5 times that of males of the same age (2). The epidemiological survey data provided by the US Preventive Medicine Working Group show that in 2020, the number of patients with osteoporosis in the population over 50 years old in the United States is expected to reach 12 million (3).

Osteoporotic fractures are the inevitable outcome of long-term and sustained bone loss, which is a major cause of death and disability among the elderly, and brings huge social and economic burdens. In aging society, one osteoporotic fracture occurs every 3 seconds in the world, and about 50% of women and 20% of men have their first osteoporotic fracture after the age of 50 (1). Due to abnormal bone metabolism, compromised bone healing, and relatively long-time immobilization for osteoporotic fractures patients, secondary bone loss and complications including pneumonia, pressure ulcers and thrombosis have brought great difficulties to clinical diagnosis and treatment. According to statistics, 21%~30% of elderly hip fracture patients die within 1 year after injury (3).

Osteoporotic fractures are often dealt with surgeries. But there are three major misconceptions currently popular among orthopedic surgeons in China: insufficient understanding of the primary disease osteoporosis, lacking concept of abnormal bone metabolism environment in osteoporosis, and underestimating bone grafting in surgery. Thus, the clinical outcome is often unsatisfactory.

The author summarized over 20 years of experience in diagnosis and treatment of osteoporotic fractures, and proposed the “Three-in-one” bone repair strategy, emphasizing the concept of vigorous anti-osteoporosis, proper bone grafting and promoting bone healing, and expects to further improve the diagnosis and treatment efficacy and the quality of life of patients.

## 2 Difficulties in Diagnosis and Treatment of Osteoporotic Fractures

Compared with fractures due to high-every trauma, osteoporotic fractures show following features: (1) It is more common in the elderly, has more complications, and is difficult to manage and recover; (2) During the fracture, immobilization or reduced exercise results in rapid secondary bone loss; (3) Common bone defects due to compression lead to difficulty in surgical reduction and stability; (4) Improper internal fixation leads to loss of internal fixation failure; (5) The risk of delayed fracture union or nonunion is higher; (6) The risk of re-fracture is significantly increased. However, there are three major misconceptions in the current clinical treatment of osteoporotic fractures.

### 2.1 Cognition Misconception: Insufficient Understanding of Osteoporosis

Osteoporotic fractures result from chronic bone loss due to osteoporosis. Abnormal bone metabolism and weakening of bone formation ability in the body substantially delay the healing of fractures. In the process of diagnosis and treatment of elderly osteoporotic fractures, orthopedic surgeons should fully pay attention to the negative effects of osteogenesis/osteoclastogenesis imbalance on fracture healing. At present, the Chinese people’s awareness of osteoporosis is generally insufficient. Among those >50 years old, the proportion of people who have undergone bone density testing is 3.7%, and the proportion of people with low bone mass is 57.4% (2). In addition, orthopedic surgeons’ attention to the bone mass and quality needs to be improved. More than 50% of patients with osteoporotic fractures have not received systematic anti-osteoporosis treatment, and only <10% of orthopedic doctors choose bone mineral density examinations for patients with first fractures.

### 2.2 Concept Misconception: Neglect of Bone Micro-Environment

Bone maintains dynamic balance coordinated by osteoclast-mediated bone resorption and osteoblast-mediated bone formation (4). Fluctuations in estrogen levels in postmenopausal women lead to excessive activation of osteoclasts, and the bone formation ability by mesenchymal stem cells (MSCs) and osteoblasts gradually decreases with aging (5). The second stage of fracture healing includes the recruitment of MSCs, cartilage callus formation, hard callus formation, and bone remodeling (6). In the case of osteoporosis, the key steps such as blood vessel ingrowth and bone remodeling are compromised, which needs to be regulated by local physical or biochemical factors (7–9). When dealing with osteoporotic fractures, orthopedic surgeons often focus on the alignment and internal fixation of the fracture, while ignore the local abnormal bone metabolism environment.

### 2.3 Technique Misconception: Ignorance of Bone Grafting

In patients with osteoporosis, the weakening of bone density and the binding force of the bone-implant interface directly leads to the decrease of screw holding, which increases the risk of internal fixation failure. Abnormal bone metabolism around the screw increases the incidence of internal implant cutting. In addition, low-strength cortical bone and high-porosity cancellous bone structures tend to form large-area bone defects when fractures occur, which also poses challenges to the initial stability of internal fixation. At present, orthopedic surgeons have not paid enough attention to the concept of bone grafting in reduction and fixation, and the stability by internal fixation is insufficient, which is likely to cause the later internal fixation failure and even a second fracture (10).

In the context of chronic bone loss and decreased bone formation ability, the healing rate of osteoporotic fracture patients decreases. Immobilization-related complications by delayed union also inhibit fracture healing. In order to break this vicious circle, orthopedic surgeons should focus on maintaining bone quality, repairing bone defect and promoting bone healing, which will systematically optimize the diagnosis and treatment of osteoporotic fractures.

## 3 “Three in One” Bone Repair Strategy

### 3.1 Core Ideology

Osteoporotic fracture is the most severe complication of osteoporosis. The treatment is to promote and accelerate fracture healing. In the past decade, 915 cases of hip fracture aged over 80 years old have been treated in the department of orthopedic trauma of the First Affiliated Hospital of PLA Naval Medical University. All patients achieved a satisfactory prognosis with the guidance of systematic and personalized treatment, including general status adjustment, appropriate implant and enhanced fracture union during the perioperative period (11).

**Figure 1:**
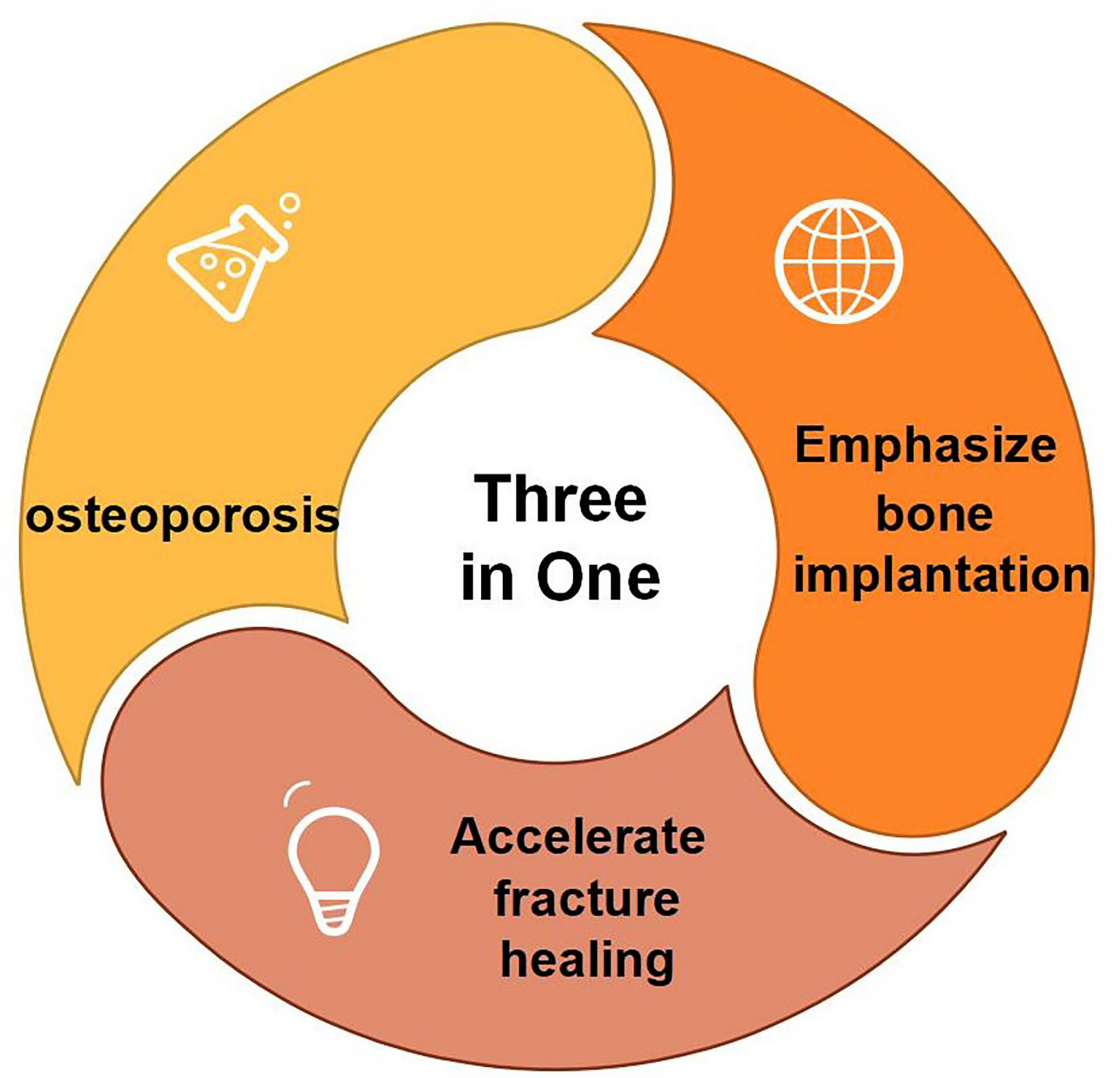
“Three in One” bone repair strategy for osteoporotic fractures. The “Three in one” strategy consists of anti-osteoporosis, bone implantation and fracture healing acceleration.

To solve this, the authors summarized the “Three in one” bone repair strategy based on clinical experiences and basic researches, focusing on the baseline bone mass, bone deficiency management and fracture union, and correcting misconceptions in cognition, concept and technique. It is believed that patients with osteoporotic fracture could obtain a better quality of life and a better prognosis through vigorous anti-osteoporosis, appropriate bone grafting and accelerated fracture healing, which are the three core matters of “Three in one” bone repair strategy (**Figure 1**).

### 3.2 Anti-Osteoporotic Fracture

#### 3.2.1 Prevent Perioperative Period Bone Loss

Intervention of perioperative period bone loss has been well recognized by surgeons all around the world (12, 13). Bone senses mechanical signals during metabolism and converts them into biological signals to adapt biomechanical requirements. During bone remodeling, bone resorption occurs at the site of less stress and bone formation occur at the site of more stress (14). Proper mechanical stimulation regulates bone metabolism through complex signaling pathways such as nerve growth factor-tyrosine kinase receptor 1 (NGF-TrkA), transforming growth factor-beta (TGF-beta). Lacking load results in rapid bone loss at rates of up to 1% per week, compared to physiological bone loss of only 1% per year in normal adults (15, 16). Rapid bone loss exacerbates osteoporosis, prolongs the duration of bed rest and restricts functional exercise, which aggravates the bone loss process in a vicious circle. The assessment of clinical osteoporosis status mainly includes symptoms, laboratory tests, imaging tests and bone density tests. Among the laboratory tests for bone metabolic indicators are mainly the bone formation marker N-terminal propeptide of type I procollagen (P1NP) and the bone resorption marker collagen type-I crosslinked C-peptide (S-CTX). Dual-energy X-ray assay (DXA) is used for bone density test. The difference of T value is used as a standard. It is usually considered that T value ≥ -1.0 SD represents normal bone mass, -1.0 SD > T value > -2.5 SD represents reduced bone mass, and T value ≤ -2.5 SD is osteoporosis, and those with fragility fracture on this basis are severe osteoporosis (1). In addition, muscle impairment and pain that significantly affect disability status in osteoporotic patients and that might be improved by anti-osteoporotic drugs, particularly those with fragility fractures (17, 18).

#### 3.2.2 Focus on Internal Fixation Stability

Factors affecting the stability of internal fixation in osteoporotic fractures mainly include screw holding force and bone healing rate (19, 20). Studies have shown that the level of T-value in DXA examination or the level of bone density in quantitative CT testing is directly related to the risk of internal fixation failure, with a significant increase in the rate of internal fixation failure in patients with local osteoporotic bone density values <95 mg/cm^3^ (19). In a study of elderly osteoporotic proximal femur fractures, the incidence of screw cut-out was found to be significantly higher in the femoral bone density value <250 mg/cm^3^ group (21). Interestingly, cortical bone thickness is a key factor affecting screw holding, and a 1 mm decrease in cortical bone thickness is associated with a subsequent decrease in screw holding force of 1000 N (or 50%) (22).

#### 3.2.3 Apply a Systematic Anti-Osteoporosis Therapy

**Figure 2:**
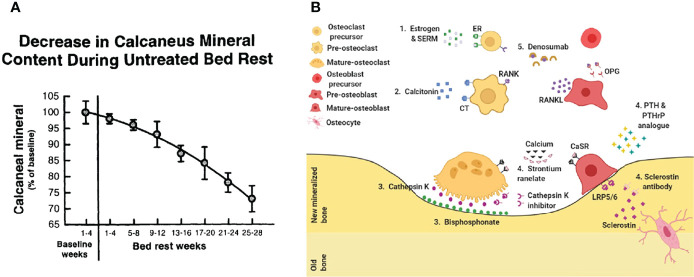
Treat osteoporosis. **(A)** Bedridden leads to accelerated bone loss (23). **(B)** Current drug treatment. 1. Estrogen & SERM, 2. Calcitonin, 3. Bisphosphonate, 4. Sclerostin antibody, 5. Denosumab (24).

Perioperative anti-osteoporosis treatment has become a consensus worldwide (**Figure 2**) (25). Perioperative anti-osteoporosis treatment mainly consists of both basic supplements and anti-osteoporotic drug therapy. The average daily intake of elemental calcium in the elderly population is approximately 400 mg, and still need an additional supplementation of about 600 mg calcium carbonate and calcium citrate (26). Active vitamin D is routinely used with calcium, guidelines and expert consensus recommend an osteoporosis treatment dose of approximately 800 to 1200 IU daily (1). As for anti-osteoporosis drugs, the first-line drugs commonly used in clinical practice are bisphosphonates (27). Bisphosphonates inhibit the bone resorption effect of osteoclasts after binding to hydroxyapatite and are more effective in the treatment of postmenopausal osteoporosis characterized by excessive osteoclast activation (28). Currently, a novel osteoclastic activity inhibitor, Denosumab, has been used clinically as a monoclonal antibody to nuclear factor-κB (NF-κB) receptor activator ligand (RANKL) that specifically inhibits osteoclasts differentiation, thereby reducing bone resorption and improving bone quality (29, 30). It is worth noting that, recent advances have shown that the nuclear factor-κB receptor-ligand (RANK-RANKL) signaling pathway promotes osteoclast maturation while inhibiting the osteogenic differentiation process of mesenchymal cells (31–33). In response to age-related osteoporosis, in which decreased osteogenic capacity is the main pathological factor, the efficacy of drugs that modulate MSC osteogenic differentiation is relatively favorable (34–36). Teriparatide, a parathyroid hormone analog, can promote new bone formation and enhance bone healing efficiency when administered intermittently at low doses (37).

### 3.3 Emphasize on Bone Grafting

#### 3.3.1 Provide Sufficient Bone Grafting

Appropriate compressive stress should be applied to promote fracture healing in accordance according to the classic AO theory (38, 39). Sufficient bone grafting is vital for anatomical reduction, stability and microenvironment improvement. Reliable initial stability and cross-sectional compressive stress are difficult to obtain because the local cancellous bone is sparse and the strength of cortical bone is low in the fracture site of patients with osteoporosis and thus bone grafting is commonly used. The intraoperative bone grafting is helpful to restore and maintain the normal anatomy and alignment of the limb and articular surface, and increase the holding force of the internal fixation. Autologous fibular segment transplantation is helpful in internal fixation of comminuted proximal humeral fractures with insufficient medial support (40). The application of artificial bone and allograft bone filling in internal fixation of tibial plateau fractures has been recognized (41, 42).

#### 3.3.2 Improve Bone Grafting Techniques

**Figure 3:**
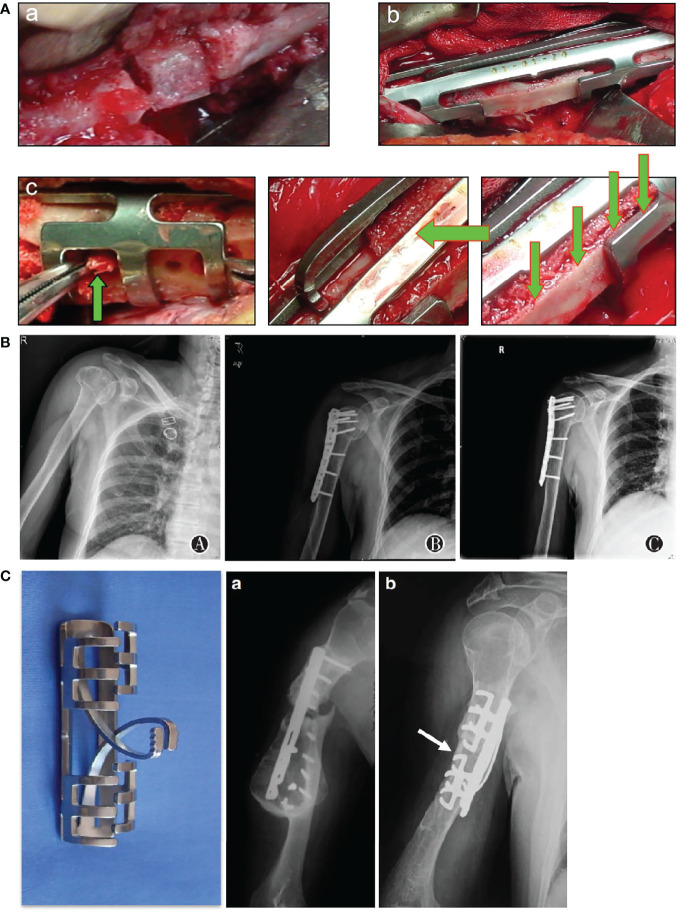
Emphasize proper internal fixation and bone implantation. **(A)** Improvement in bone grafting method. a cushion method; b splint method; c seeding method (45). **(B)** A 74-year-old patient with proximal humerus fractures treated with open reduction and internal fixation with allograft bone graftings. a preoperative image; b immediate postoperative image; c 12 months after surgery (46). **(C)**a. A 55-year-old male patient with humeral shaft fracture, nonunion occurred 6 months after internal fixation using a dynamic compression plate due to screw breakage; **(b)** Four months after implantation of shape memory Ni-Ti alloy swan-like bone connector fixation and autologous bone grafting (45).

Compared with conventional fractures by high energy trauma, osteoporotic fractures have larger bone defects and are more difficult to maintain stability after internal fixation, which laid higher requirements for the volume and technology of bone grafting materials (43). Autologous bone is the best bone grafting material with low immunogenicity, good bone induction and mechanical conduction. Nevertheless, a large number of autologous bone increase risks of infection, pain and even iatrogenic fracture (44). To improve the utilization rate of autologous bone, clinicians are supposed to take some measures to promote the success of bone grafting which requires reliable fixation and sufficient blood supply. The cushion method enhances the holding force of the screw end and the stability of internal fixation by inserting the screw into the contralateral cortical bone. In order to fully repair bone defects and achieve early postoperative functional exercise and rehabilitation, various bone grafting skills can be used when dealing with huge bone defects (**Figure 3**).

#### 3.3.3 Develop New Grafting Biomaterials

Currently, a large number of available allogeneic bone and artificial bone materials in clinical application can be selected by orthopedic surgeons based on specific demands (47, 48). Biomaterials could promote fracture healing by stimulating the bone formation ability of skeletal stem cells as well as providing tissue growing scaffolds. The new biological mesoporous materials are absorbed and degraded by osteoclasts, followed by the formation of hydroxyapatite and bone matrix mineralization (49). Bone cement reinforcement technology can reduce the occurrence of internal fixation loosening and reduction loss in the decompression, reduction and pedicle screw internal fixation of osteoporotic vertebral compression fracture (50). Non-self-heating phosphate bone cement materials is worthy of attention (51). The new inorganic mesoporous microsphere material can promote the growth and effective integration of bone tissue locally, which promote fracture healing while realizing mechanical filling (52).

### 3.4 Accelerate Fracture Healing

#### 3.4.1 Select Appropriate Internal Fixations

The stability of the traditional internal fixation depends on the friction between the internal fixation and the bones (53). However, the stability is significantly compromised in osteoporosis, and complications such as screw cutting and loosening are often. The principle of choice of internal fixation for osteoporotic fractures is to increase stability and preserve bone mass. In terms of plates selection, the locking plate is preferred due to less dependence on the holding force of the screw. The multi-angle fixation based on the anatomical shape further increases the stability. When dealing with fractures such as proximal humeral fractures, the cement intensifying technology of the screw channel can effectively increase the screw holding force, and relatively reduce the risk of humeral head collapse and screw cutting (54, 55). When dealing with non-load-bearing diaphyseal fractures, the new shape memory alloy bone connector provides continuous and effective axial stress stimulation for the fracture end while resisting shear, bending and rotation, which is conducive to fracture healing (56).

#### 3.4.2 Apply Bioactive Factors

Apart from the nature of the biomaterial itself, the biologically active factors loaded also play an important role in the process of fracture healing (57, 58). Biomaterials loaded with bone morphogenetic protein-2 (BMP-2) can promote osteogenic differentiation and bone repair (59). In addition, the problem that the drug concentration continues to decrease when the carrier releases biological factors locally cannot be avoided, and new bone-targeted drug carriers such as exosomes show considerable therapeutic potential, and the local biological microenvironment regulation of fractures can be achieved through targeted drug delivery. The effect of metal ions on local stimulation of fracture healing has been confirmed. The novel magnesium-based bone cement material can activate the osteoblast signal pathway while filling the defect to improve the bone repair effect (60, 61).

#### 3.4.3 Guide Postoperative Rehabilitation

The functional recovery of patients with osteoporotic fractures is restricted by multiple factors, and the reduced stability of internal fixation renders higher risks to functional exercise. Appropriate rehabilitation aids allow the fracture site to withstand less shear stress and improve safety (62, 63). Under the concept of accelerating bone healing, perioperative functional exercises protected by rehabilitation aids are essential to break the vicious circle and accelerate bone healing. The rigid support provided by the spinal orthosis can assist patients with vertebral compression fractures to walk after surgery, reducing the load and pain of the spine. Foot rehabilitation aids have good results in postoperative applications of calcaneal fractures and ankle fractures (64). Some elderly patients with osteoporotic fractures have more complications and relatively high surgical risks. When choosing non-surgical treatment, attention should be paid to weight-free exercise under the protection of rehabilitation aids to speed up the process of bone healing.

## 4 Inspirations by Three in One Strategy

### 4.1 Explore Pathogenic Mechanisms of Osteoporosis

The pathogenic mechanism is the basis of developing novel drugs or interventions. The understanding of osteoclastogenesis has led to the development and application of bisphosphonates and RANKL inhibitors. Although the osteoclasts inhibiting strategies have been used in clinical experience for decades, there are still side effects unsolved. For example, untypical fractures and osteonecrosis brought by bisphosphonates might be well handled by latest candidate of anti-osteoclastogenesis drug (65). So far, interesting progresses have been reported by surgeons and researchers.

#### 4.1.1 L-Plastin Mediates Osteoclasts Fusion and Bone Resorption

**Figure 4:**
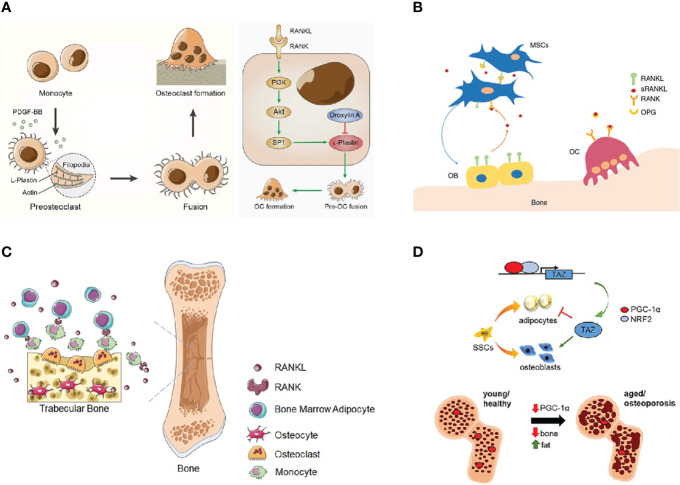
Explore pathogenic mechanism of osteoporosis. **(A)** Targeting L-plastin prevents pre-osteoclasts fusion and promotes PDGF-BB secretion (35). **(B)** RANKL signaling inhibits osteoblastic differentiation of BMSCs (31). **(C)** Bone marrow adipocyte-derived RANKL controls trabecular osteoclastogenesis (66). **(D)** PGC-1α controls skeletal stem cell fate and bone-fat balance in osteoporosis and skeletal aging (67).

The actin-bundling protein L-plastin (LPL) mediates the resorption activity of osteoclasts, but its therapeutic potential in pathological bone loss remains unexplored. Here, we report that LPL regulates osteoclasts fusion, and targeting LPL serves as a novel anabolic therapy for pathological bone loss. (Targeting actin-bundling protein L-plastin as an anabolic therapy for bone loss (**Figure 4A**) (35).

#### 4.1.2 RANKL Signaling Negatively Regulate Osteogenic Differentiation of BMSC

RANKL signaling is essential for osteoclastogenesis (68). Its role in osteoblastic differentiation and bone formation is unknown. Our results reveal that RANKL signaling regulates both osteoclasts and osteoblasts by inhibition of osteogenic differentiation of BMSCs and promotion of osteoclastogenesis. This study interprets the underlying mechanisms of unexpected bone formation by RANKL blocking and explains the sustained bone volume increase among patients taking RANKL antibodies (**Figure 4B**) (31).

#### 4.1.3 RANKL From Bone Marrow Adipocytes Controls Bone Resorption

Receptor activator of NF-κB ligand (RANKL) is essential for osteoclast formation and bone remodeling (69, 70). Nevertheless, the cellular source of RANKL for osteoclastogenesis has not been fully uncovered. BM adipose lineage cells therefore represent an essential source of RANKL for the formation of trabecula osteoclasts and resorption of cancellous bone during remodeling under physiological and pathological conditions. Targeting bone marrow adiposity is a promising way of preventing pathological bone loss (**Figure 4C, D**) (66, 67).

### 4.2 Develop Bone Grafting Biomaterials

#### 4.2.1 Target Osteoclasts to Inhibit Bone Resorption

**Figure 5:**
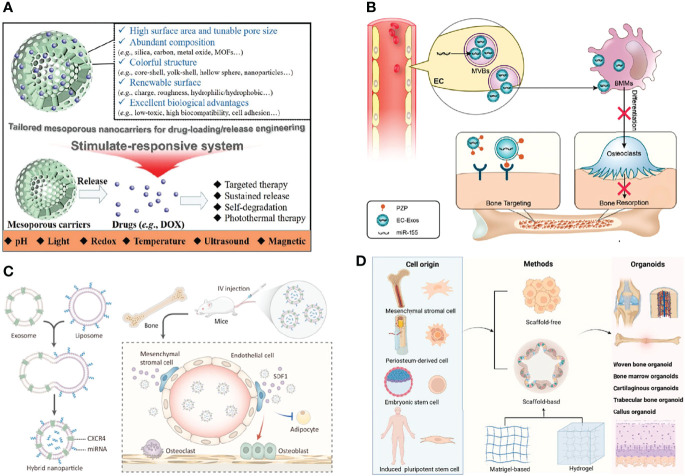
Develop bone grafting biomaterials. **(A)** The advantages of tailored mesoporous nanocarriers (71). **(B)** Endothelial cell-secreted exosomes could be internalized by bone marrow-derived macrophages and inhibit osteoclastogenesis (61). **(C)** The bone-targeted hybrid nanoparticle that promotes osteoclastogenesis and inhibits adipogenesis of BMSCs (72). **(D)** The potential applications of bone organoid (73).

Various mesoporous biomaterials are exploited and applied as efficient nanocarriers to loading drugs by virtue of their large surface area, high porosity, and prominent biocompatibility. Herein, recent progress and assembly mechanisms on mesoporous inorganic biomaterials are summarized systematically, and typical functionalization methods for nanocarriers are also discussed in depth. Particularly, structure-activity relationship and the effect of physicochemical parameters of mesoporous biomaterials in DDS application are overviewed. As one of the important development directions, advanced stimuli-responsive DDSs are highlighted. Finally, the prospect of mesoporous biomaterials in disease therapeutics is stated, and it will open a new stage for the development of mesoporous nanocarriers (**Figure 5A**) (71).

Though the relationship between vascular and bone homeostasis has been recognized recently, the role of vascular endothelial cell (EC)-secreted exosomes (EC-Exos) in bone homeostasis is not well understood. Herein, we found that EC-Exos inhibit osteoclast activity *in vitro* and inhibit osteoporosis in an ovariectomized mouse model. Our findings suggest that EC-Exos may be utilized as a bone targeting and nontoxic nanomedicine for the treatment of bone resorption disorders (**Figure 5B**) (61).

#### 4.2.2 Target Mesenchymal Stem Cell Niches to Promote Osteoblastic Bone Formation

The differentiation shift from osteogenesis to adipogenesis of bone marrow mesenchymal stem cells (BMSCs) characterizes many pathological bone loss conditions (74, 75). Stromal cell-derived factor-1 (SDF1) is highly enriched in the bone marrow for C-X-C motif chemokine receptor 4 (CXCR4)-positive hematopoietic stem cell (HSC) homing and tumor bone metastasis. In this study, we fused CXCR4+ exosomes with liposomes carrying antagomir-188 to produce hybrid nanoparticles (NPs). The hybrid NPs promoted osteogenesis and inhibited adipogenesis of BMSCs and thereby reversed age-related trabecular bone loss. Taken together, this study presents a novel way to obtain bone-targeted exosomes *via* surface display of CXCR4 and a promising anabolic therapeutic approach for age-related bone loss (**Figure 5C**) (72). Based on the above study, we summarized strategies of engineered extracellular vesicles for bone therapy (76, 77).

#### 4.2.3 Build Bone Organoids to Promote Defect Repair

Bone regeneration is a key issue in the clinics. Bone tissue engineering technology provides various types of functional scaffold materials and seed cells for bone repair (78, 79). However, the bone metabolism mechanism is complicated. Organoids as a new concept, which is built *in vitro* with the help of tissue engineering technology, can simulate the complex biological functions of organs *in vivo*. Once proposed, it shows broad application prospects in the research of organ development, drug screening, mechanism study, and so on. As a complex and special organ, bone organoid construction itself is quite challenging. In this study, we introduced the characteristics of bone microenvironment, the concept of organoids, focused on the research progress of bone organoids, and proposed the strategies for bone organoid construction, study direction, and application prospects (**Figure 5D**) (73).

## 5 Outcomes of Implementation of the Three in One Bone Repair Strategy

“Three in one” bone repair strategy reflects and summarizes the wisdom of dealing with osteoporotic fractures. Wang et al. reported that bisphosphonate and non-bisphosphonate medications for osteoporosis were significantly associated with decreased mortality after fragility hip fracture (80). Tai et al. showed that anti-osteoporosis treatment after hip fracture is associated with lower all-cause mortality (81). A longer duration of treatment was also associated with lower mortality. Besides, studies reported that muscle impairment and pain significantly affecting disability in osteoporotic patients might be improved by anti-osteoporotic drugs, particularly those with fragility fractures (17, 18). Although bone morphogenetic proteins (BMPs) show their potential roles in promoting fracture healing by increasing osteoblastic formation in osteoporotic patients, there has been no current published clinical data (82). Limited evidence of animal studies suggests that BMPs could stimulate fracture healing in ovariectomized rats (83). Therefore, the therapeutic effects of BMPs in osteoporotic fractures should be verified with further clinical trials. As for rehabilitation, although currently there has been no conclusion on the intensity, frequency and duration of postoperative rehabilitation, many studies reported positive effects of early activity and rehabilitation on functional recovery (84). These studies are somewhat consistent with the “Three in one” strategy stressing on anti-osteoporosis therapy, intraoperative bone grafting and rehabilitation, although reported independently.

Based on the “Three in one” bone repair strategy, we drafted three experts on important clinical problems in the treatment of osteoporotic fractures: Expert consensus on bone repair strategies for osteoporotic fractures in China (2019) (Consensus 1) (85), Chinese expert consensus on perioperative management of osteoporotic fractures (2018) (Consensus 2) (86), Expert consensus on lower limp periarticular osteoporotic fractures (Consensus 3 and 4) (2020) (87, 88).

We believe that the perioperative period is critical for orthopedic surgeons to manage osteoporosis and osteoporotic fractures. In view of the current misconceptions in osteoporotic fracture treatment, our team published Consensus 2, which elaborates the surgical protocols, rehabilitation time points and complication management of osteoporotic fracture treatment, and emphasizes the necessity and strategies of perioperative anti-osteoporosis treatment widely recognized in clinical practices (86). With regards to technical misconceptions, the team further established Consensus 1, which focused on the features of bone defects, clinical bone repair materials and repair strategies for common fractures based on Consensus 2, to promote the clinical application of proper bone grafting for osteoporotic fractures (85). As for the treatment of the most prevalent fractures, especially hip and vertebral fracture, the team developed Consensus 3, aiming to summarize the experience of clinical treatment of osteoporotic fracture to provide a systemic rehabilitation treatment plan, and promote the individualized fracture management (2020; 2020).

## 6 Conclusion and Prospect

Osteoporotic fracture is not just a simple local fracture, but a manifestation of abnormal systemic bone metabolism. In the clinical diagnosis and treatment of osteoporotic fractures, the lack of understanding of the primary disease, bone metabolism environment, and the technical underestimation of bone grafting leads to unsatisfactory prognosis.

Osteoporotic fractures heal slowly, and long-term bed rest accelerates bone loss, forming a vicious circle. The key to breaking the vicious circle is to accelerate bone healing. China has entered an aging society. With the aging of the population and the improvement of quality of life, elderly patients with osteoporotic fractures urgently need faster and better treatment. Orthopedic surgeons need to understand the abnormal state of bone metabolism, choose the appropriate implants for fracture fixation, and appropriately use bone healing materials and rehabilitation aids to accelerate fracture healing under the concept of “Three in One” bone repair strategy. The purpose is to improve the clinical prognosis of elderly patients with osteoporotic fractures.

## Author Contributions

XC, YH, and ZG contributed equally to this work. JS conceived the manuscript. XC, YH and ZG wrote the manuscript. JS and XC reviewed and edited the manuscript. All authors listed have made a substantial, direct and intellectual contribution to work, and approved it for publication.

## Funding

This project was supported by the National Key R&D Program of China (2018YFC2001500); National Natural Science Foundation of China (82172098, 81871099, 91749204, 81771491).

## Conflict of Interest

The authors declare that the research was conducted in the absence of any commercial or financial relationships that could be construed as a potential conflict of interest.

## Publisher’s Note

All claims expressed in this article are solely those of the authors and do not necessarily represent those of their affiliated organizations, or those of the publisher, the editors and the reviewers. Any product that may be evaluated in this article, or claim that may be made by its manufacturer, is not guaranteed or endorsed by the publisher.
